# Exploration on the potential efficacy and mechanism of methyl salicylate glycosides in the treatment of schizophrenia based on bioinformatics, molecular docking and dynamics simulation

**DOI:** 10.1038/s41537-024-00484-y

**Published:** 2024-07-17

**Authors:** Xiuhuan Wang, Jiamu Ma, Ying Dong, Xueyang Ren, Ruoming Li, Guigang Yang, Gaimei She, Yunlong Tan, Song Chen

**Affiliations:** 1 Peking University HuiLongGuan Clinical Medical School, Beijing HuiLongGuan Hospital, Beijing, 100096 PR China; 2 School of Chinese Materia Medica, Beijing University of Chinese Medicine, Beijing, 102488 PR China

**Keywords:** Biomarkers, Schizophrenia, Drug delivery, Pharmacology, Target identification

## Abstract

The etiological and therapeutic complexities of schizophrenia (SCZ) persist, prompting exploration of anti-inflammatory therapy as a potential treatment approach. Methyl salicylate glycosides (MSGs), possessing a structural parent nucleus akin to aspirin, are being investigated for their therapeutic potential in schizophrenia. Utilizing bioinformation mining, network pharmacology, molecular docking and dynamics simulation, the potential value and mechanism of MSGs (including MSTG-A, MSTG-B, and Gaultherin) in the treatment of SCZ, as well as the underlying pathogenesis of the disorder, were examined. 581 differentially expressed genes related to SCZ were identified in patients and healthy individuals, with 349 up-regulated genes and 232 down-regulated genes. 29 core targets were characterized by protein-protein interaction (PPI) network, with the top 10 core targets being BDNF, VEGFA, PVALB, KCNA1, GRIN2A, ATP2B2, KCNA2, APOE, PPARGC1A and SCN1A. The pathogenesis of SCZ primarily involves cAMP signaling, neurodegenerative diseases and other pathways, as well as regulation of ion transmembrane transport. Molecular docking analysis revealed that the three candidates exhibited binding activity with certain targets with binding affinities ranging from −4.7 to −109.2 kcal/mol. MSTG-A, MSTG-B and Gaultherin show promise for use in the treatment of SCZ, potentially through their ability to modulate the expression of multiple genes involved in synaptic structure and function, ion transport, energy metabolism. Molecular dynamics simulation revealed good binding abilities between MSTG-A, MSTG-B, Gaultherin and ATP2B2. It suggests new avenues for further investigation in this area.

## Introduction

Schizophrenia (SCZ) is a debilitating disorder involving multiple types of brain dysfunction, characterized by hyperactivity that can lead to positive symptoms such as hallucinations and delusions, and negative symptoms such as cognitive impairment, poor thinking, apathy, and behavioral withdrawal^1,2^. Genetic factors play a role in many cases, with an 80% heritability of risk, decreasing by 50% with each degree of familial relationship^3^. Environmental factors, including chronic stress, physical or emotional trauma during childhood, may also increase the risk of SCZ^4^. At present, some other aspects including a decrease in social and occupational functioning, especially an inevitable side effect of existing therapeutic drugs, both contribute to both the aetiology and treatment aspects of schizophrenia remain challenging to study.

Since the early 1990s, many opinions and perceptions have been proposed to explain the onset of SCZ in an immunocompetent manner, such as the macrophage-T lymphocyte theory, the general inflammation hypothesis, the immune hypothesis, autoimmunity hypothesis, the microglia hypothesis, and the immune-inflammatory balance hypothesis^5^. They have become one of the strongest arguments in favor of an autoimmune and/or immunoinflammatory origin of SCZ. Inflammation and immune regulation play an important role in the development and maintenance of SCZ^6^. Inflammation and immune dysfunction have been reported to contribute to the cognitive, negative, and positive symptoms of SCZ^6,7^. Inflammatory processes associated with persistent/chronic infections have been implicated in psychiatric disorders^8^. Aspirin, also known as acetylsalicylic acid, is a non-steroidal anti-inflammatory drug with obvious anti-inflammatory and analgesic effects. It is used in the treatment of acute and chronic rheumatic diseases, the early treatment and prevention of cardiovascular diseases, and the cerebrovascular diseases. Aspirin has properties that inhibit the proinflammatory state of the brain^9^, and may reduce the risk of cardiovascular disease and mortality in patients with SCZ^10^. Drugs are being investigated for their role as adjunctive or monotherapy in the treatment of SCZ. Hormone therapy, antioxidants, ω3 fatty acids, and other anti-inflammatory agents such as minocycline, have shown significant effects in reducing total score, positive and negative scoring symptoms, and overall functioning in patients with schizophrenia^11^. All of the above studies have provided scientific evidence and illustration that anti-inflammatory or immunotherapeutic strategies are relevant for SCZ.

The multi-component and multi-target action characteristics of Traditional Chinese Medicine (TCM) and Ethnic Medicine (EM), as well as the advantage of low toxicity and side effects of them have been of great interest to the majority of researchers. Dianbaizhu is an EM herb that we have been researching for a long time, and it is commonly used to treat rheumatoid arthritis in the southwest of our country^12^. At the early stage, with the support of in the two National Natural Science Foundation of China (NSFC) projects, our team conducted a series of studies, including screening of medicinal parts, chemical composition, enrichment of active parts, intestinal absorption characteristics in situ and in vitro, pharmacokinetics and exploration of anti-rheumatic mechanism^13^. We determined its medicinal active part and isolated its main active ingredients (methyl salicylate glycoside components, MSGs), named as MSTG-A, MSTG-B and Gaultherin, respectively, all of which have been reported to have anti-inflammatory and analgesic effects^14,15^. Their chemical structure differs only in the position and number of glucose and xylose substitutions, and they have been shown to interconvert in vivo and in vitro^16,17^. Eventually, they are present in the animal in the form of their metabolites, salicylic acid or methyl salicylate^17,18^. Both their prototype and metabolite share the same parent structural core, which is similar to aspirin. Based on the principle of similar structure and similar properties, together with the research strategy of anti-inflammatory immune regulation, it has been speculated that the three methyl salicylate glycosides may have good therapeutic activity in SCZ.

**Fig. 1:**
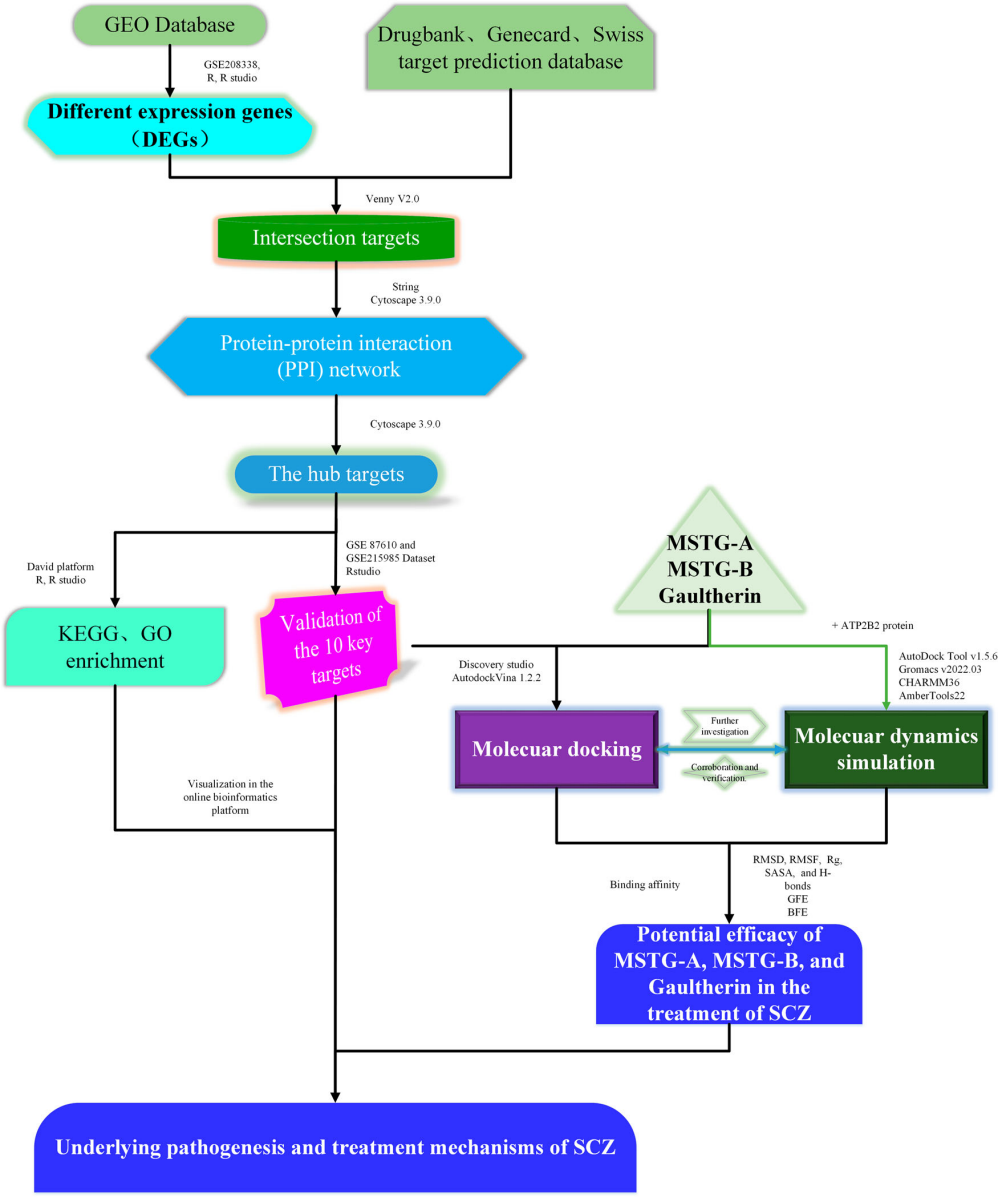
A schematic diagram to reveal the pathogenesis mechanisms and treatment efficacy of SCZ.

Do they have such activity and what is the underlying mechanism of this activity? According to the research purpose, we mainly adopted bioinformatic data mining, network pharmacology and molecular docking technology as the key technologies of this study. Accordingly, the mining of differentially expressed genes (DEGs) between SCZ patients and healthy controls based on GEO data, the screening and verification of SCZ key targets based on network pharmacology technology, and the exploration of the potential efficacy and mechanism of MSGs in the treatment of SCZ based on molecular docking and dynamics simulation technology were carried out respectively. This is the schematic procedure for this study (Fig. 1).

## Materials and methods

### Data acquisition and standardization

Gene Expression Omnibus (GEO) Dataset GEO series GSE studies were gathered. A study on SCZ was conducted using bioinformatics methods, with some modifications to the reported methodology^19^. GSE chip data were retrieved from the GEO database (https://www.ncbi.nlm.nih.gov/geo/) using the keywords “schizophrenia” and “Homo sapiens”. Inclusion criteria included: (1) the study must encompass patients with SCZ and normal controls; (2) Findings from multiple studies have clearly implicated pathology of the dorsolateral prefrontal cortex as playing a central role in the pathophysiology of SCZ, particularly with regard to key cognitive features such as deficits in working memory and cognitive control. In order to get closer to the essential questions that respond to the onset of SCZ, the detection of gene expression profiles in postmortem dorsolateral prefrontal cortex (DLPFC) as a selection criterion was included in this study. GSE208338 datasets were finally selected and downloaded from the publicly available databases. GSE208338 consisting of 192 SCZ (SCZ group) and 170 normal controls (CTL group), for further analysis. The probe IDs was were annotated and converted to a standard gene name (gene symbol) by applying the platform file (GPL5188) and R software^20^. After deleting the missing values, a standardized gene expression matrix was obtained for the next analysis.

### Identification of DEGs

To identify DEGs for the GSE208338 datasets between SCZ and normal controls, normalization of the common gene expression data was performed using the limma package in R. The values of |logFC | > (mean | logFC | + 2 × SD | logFC |) and *P* < 0.05 were considered as DEGs^19,21^. The heatmap and volcano of DEGs were generated using the “ggplot” packages of the R Studio 4.3.3 software.

### Construction of molecular networks and mining of key targets

A Venn diagram of SCZ-related genes and DEGs was created on the web of Venny 2.1.0 (https://bioinfogp.cnb.csic.es/tools/venny/index.html)^22^. The genes that overlapped in the Venn diagram were considered as candidate key genes of SCZ. The protein-protein interaction (PPI) network of the hub genes was constructed in the STRING database (https://www.string-db.org/) and Cytoscape 3.9.0 software.

The degree value (DV), betweenness centrality value (BCV) and closeness centrality value (CCV) of the PPI network topology features were calculated. The targets, whose DV, BCV and CCV values of each target were greater than the median values, respectively, were reckoned as the key targets.

### Functional enrichment analysis

We then analyzed the 29 core targets for enrichment in KEGG pathways and GO according to the published method. The gene symbol of the DEGs was converted to Entrez ID. The enrichment analysis of the gene ontology (GO) and the Kyoto Encyclopedia of Genes and Genomes (KEGG) was performed through the David database (https://david.ncifcrf.gov/tools.jsp)^23^. *P* value < 0.01 and *FDR* (false discovery rate) < 0.05 were set as a significant enrichment criterion. The bubble chart, bar with color gradient of significantly altered targets were plotted using the online bioinformatics platform (http://www.bioinformatics.com.cn).

### Validation of hub genes (top 10) and evaluation of SCZ biomarkers

Two additional GSE data (GES87610 and GSE215985) were screened and obtained from the GEO database according to the same method as the GSE208338. Combining the study results with the reported literature, the top 10 key genes were also simultaneously validated from GES87610 and GSE215985. The DEGs matrixes of GES87610 and GSE215985 data were obtained and compared with those of GSE208338. Compared with GSE208338, the accuracy of the obtained data was verified by assessing the consistency of the up or down trend of the expression of these top 10 targets.

### Molecular docking

This molecular docking protocol with a litter modification was performed according to our previously published literature^24^. Briefly, the 2D structures of the ligand compounds (MSTG-A, MSTG-B and Gaultherin) were downloaded using the PubChem database Explore Chemistry (https://pubchem.ncbi.nlm.nih.gov) and saved in “.sdf” format. Protein crystal structures were downloaded from the Protein Data Bank (PDB) database (http://www.rcsb.org/). We selected Homo sapiens crystal structures, X-ray diffraction or solution NMR, the refinement resolution < 3.30, and the other options were set as defaults. The crystal structure of the key genes, including BDNF (PDB ID, 1BND; resolution, 2.30 Å), VEGFA (PDB ID, 6ZFL; resolution, 1.60 Å), PVALB (PDB ID, 1RK9 and 1RWY; resolution, 1.05 Å), KCNA1 (PDB ID, 1EXB; resolution, 2.10 Å), GRIN2A (PDB ID, 5H8Q; resolution, 1.90 Å), ATP2B2 (PDB ID, 2KNE), KCNA2 (PDB ID, 2R9R; resolution, 2.40 Å), APOE(PDB ID, 7FCR; resolution, 1.40 Å), PPARGC1A(PDB ID, 6W9L; resolution, 1.45 Å) and SCN1A(PDB ID, 7DTD; resolution, 3.30 Å), were downloaded in “.pdb” format, respectively. The crystal structure was pre-processed and docked using the online platform (https://www.dockeasy.cn/DockCompound) and Discovery studio (DS) software. Routine processing mainly involved the removal of water molecules, addition of polar hydrogen atoms and conformation optimization, visualization, etc. MSTG-A, MSTG-B and Gaultherin were prepared by energy minimization. Molecular docking studies were carried out utilizing Autodock Vina 1.2.2 (http://autodock.scripps.edu/) from the Home for researchers platform (https://www.home-for-researchers.com/#/). The grid box was centered to cover the domain of each protein and to accommodate free molecular movement. The grid box was set to 30 Å × 30 Å × 30 Å, and the grid point spacing was 0.05 nm^25^. We determined the docking binding affinity (kcal/mol) of the small molecule ligand and the receptor protein. Taking the affinity as an evaluation index, the higher the absolute value of the affinity, the more stable the binding between the ligand and the receptor^26^. Compounds were considered to have potential anti-SCZ activity if the binding affinity values were lower than the threshold value (−4 kcal/mol)^27^.

### Molecular dynamics simulation (MDS)

MDS is a fundamental tool for elucidating the binding affinity and stability of small molecules-targets complex. To confirm the ligand–receptor binding stability, the polydatin-target protein complex (MSTG-A-ATP2B2, MSTG-B-ATP2B2, Gaultherin-ATP2B2) with the highest absolute binding free energy in molecular docking was selected for MDS by Gromacs v2022.03 software and CHARMM36^28–30^. Refer to the method in the literature, the specific process and parameters were as follows: (1) For reasons of computational time, in this section, 2KNE (PDB ID) was cleaned firstly to be a pureed 3D protein structure for carrying out molecular docking with 3 small molecules; then the “pdb” format of the three complexes were converted to “gro” format, which was regarded as the initial structure of the MDS. (2) The Generalised Amber Force Field (GAFF) force field was added to the small molecules by using AmberTools22 software^31^, and the potential data will be added to the small molecules using Gaussian 16 W for hydrogenation, root mean square deviation (RESP) calculation. (3) Three-point transferable intermolecular potential (TIP3P) was chosen to solubilize the complexes and the protein atoms were at least 1.2 nm (12 Å) away from the closest distance from the edge of the water box^32^, and by the addition of appropriate amounts of Na^+^ and Cl^-^ to neutralize the simulating the system charge (concentration: 0.154 M). (4) Energy minimization (EM) was performed using the Steepest descent algorithm (SDA)^33^. (5) The solutes were confined in an isothermal isotropic (NVT) systematic, the system was slowly heated from 0 K to 300 K; and it were equilibrated in an isothermal isobaric (NPT) systematic at a temperature of 300 K and a pressure of 1 Bar. (6) aThe complexes were subjected to MDS for 100 ns time; the simulation trajectories were saved for subsequent analyses. Based on the results of the MDS, we calculated the values of root mean square deviation (RMSD), root mean square fluctuation (RMSF), radius of rotation (Rg), solution accessible surface area (SASA), and numbers of hydrogen bonds (H-bonds). The Gibbs free energy (GFE) is calculated using the “g_sham” and “xpm2txt.py” scripts built into the Gromacs v2022.03 software. The “MMPBSA.py v.16.0” script was applied to calculate molecular mechanics/Poisson-Boltzmann surface area (MM/PBSA) for obtaining the binding free energy (BFE) of the 3 components and ATP2B2^34^. The lower the BFE value, the more stable the complex.

## Results

### Identification of DGEs based on GSE208338

**Fig. 2:**
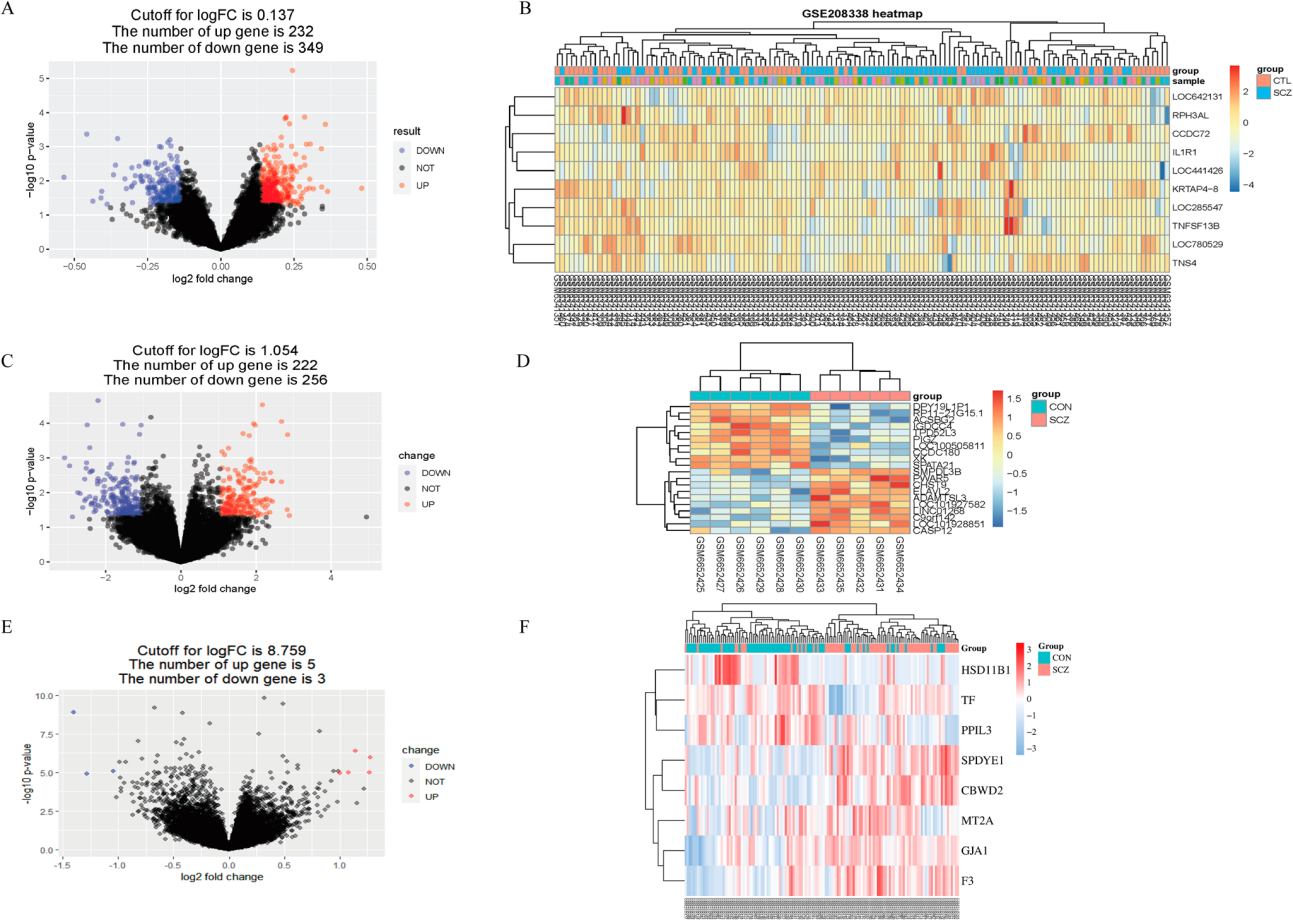
Identification of DEGs. Volcano and heatmaps of GSE208338, GSE87610 and GSE215985. **A** Volcano plot of the distribution of DEGs in SCZ and CTL group of GSE208338, upregulated expression and downregulated expression were exhibited with red dots and bluish violet dots, respectively; gray dots presented not significant expression. **B** Heatmap of the top 10 downregulated and upregulated DEGs of GSE208338. **C** Volcano plot of the distribution of DEGs in SCZ and CTL group of GSE87610. **D** Heatmap of the top 20 downregulated and upregulated DEGs of GSE87610. **E** Volcano plot of the distribution of DEGs in SCZ and CTL group of GSE215985. Not significant expressions DEGs were indicated in gray dots. **F** Heatmap of the top 10 downregulated and upregulated DEGs of GSE215985.

The GSE208338 chip was screened and analysed using the GEO database and R Studio software. To mean | logFC | + 2 SD | logFC | (0.137) and *P* < 0.05 was selected as the critical value to screen for DGEs in this dataset. After R analysis, a total of 581 DGEs were obtained in SCZ patients and healthy individuals, including 349 up-regulated genes and 232 down-regulated genes. The volcano map and heatmap were displayed as shown in Fig. 2A, B.

### Common target PPI network analysis and selection of the key targets

**Fig. 3:**
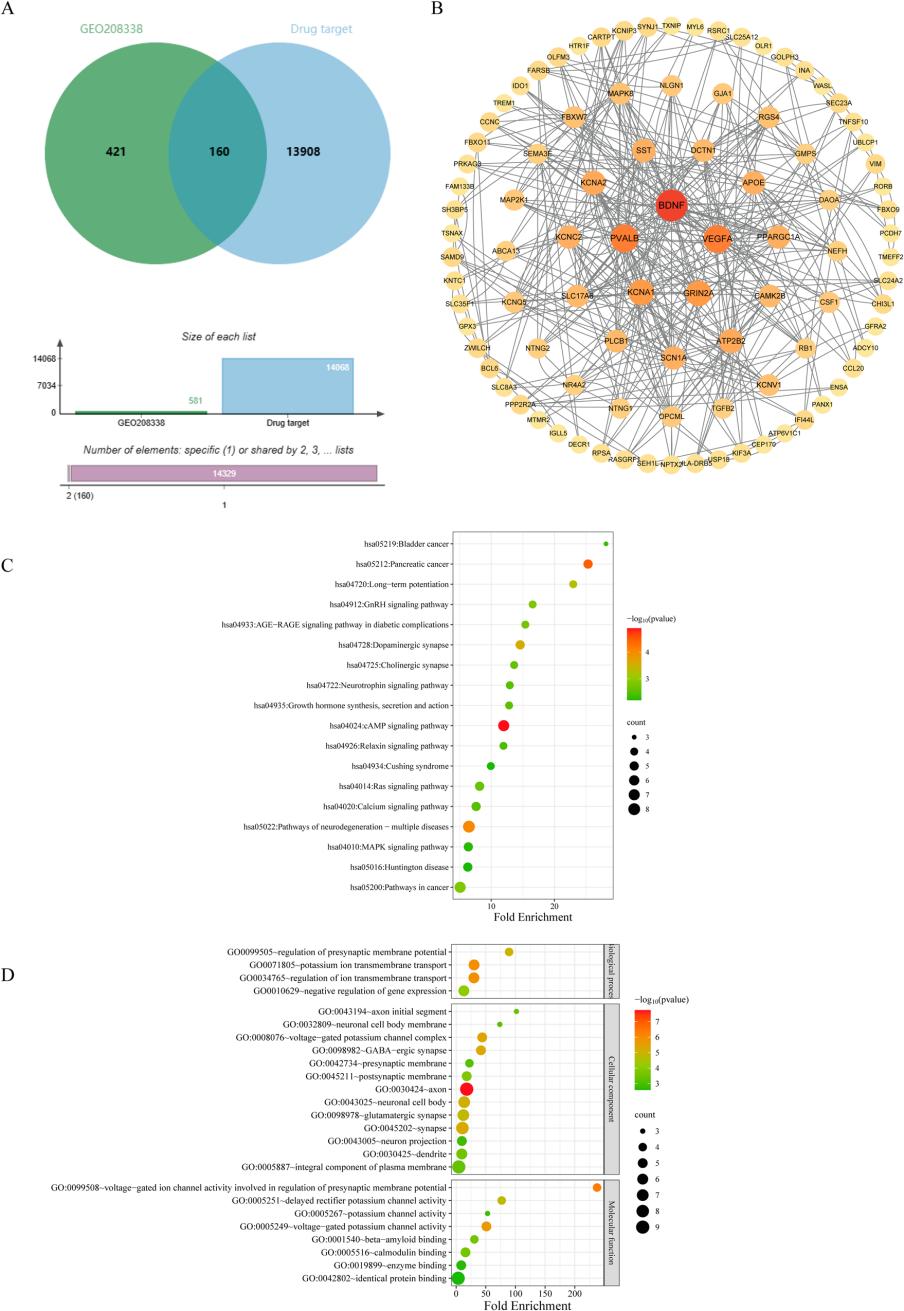
The Venn, network analysis and enrichment analysis of targets involved in SCZ. **A** The relationship of DEGs of GSE208338 and drug targets. There are 160 overlapping targets between GEO208338 and drug targets. **B** PPI network of 160 common targets. **C** KEGG pathway analyses of the 29 hub targets. The vertical and horizontal axes represent the pathway name and enrichment factors, respectively. The size of the dots demonstrates the number of targets enriched. The color of the circular dots represents the -log_10_(*p*value), and red to green indicate -log_10_(*p*value) from small to large. **D** GO analysis of 29 hub targets. The vertical axis is the name of the GO items, and the horizontal axis the enrichment factor. The size of the points indicates the number of the targets enriched. The color of the dot represents the -log_10_(*p*value), and red to green represent -log_10_(*p*value) from small to large.

A total of 15280 SCZ disease targets were obtained from the three databases, and 14068 therapeutic drug targets were obtained after screening and duplication removal. Venn diagram was plotted as exhibited in Fig. 3A. In our study, the PPI network including 160 overlapping proteins, was established to appraise the alterations in cellular functions and processes of SCZ patients. Detailed information is provided in Supplementary Table S1. 160 intersection targets were imported into the String platform to achieve the PPI network. The visualization and topology analysis were performed by using Cytoscape (version 3.9.0). The combined score and DV were used to evaluate the size of edges and nodes, the results showed that this network contained 42 nodes and 125 edges (Fig. 3B). The median values of DV, BCV and CCV were 6, 3.97 × 10^−2^ and 0.04, respectively. The three corresponding indices that were higher than the above critical value was regarded as the main nodes. A total of 29 core targets were obtained by screening the topological characteristics including BCC, BCV and CCV values. The top 10 targets, including BDNF, VEGFA, PVALB, KCNA1, GRIN2A, ATP2B2, KCNA2, APOE, PPARGC1A, SCN1A (Table 1), were finally selected as the core genes finally. It is suggested that these 29 targets are more likely to be the core targets inducing the development of SCZ. As a result, these 10 hub nodes were selected for the next GO and KEGG pathway analyses.

**Table 1:** The topological parameters of the 10 key targets.

Target name	Gene name	BCV	CCV	DV	Eccentricity
Brain-derived neurotrophic factor	BDNF	0.339	0.41	38	5
Vascular endothelial growth factor A	VEGFA	0.228	0.383	28	5
Parvalbumin	PVALB	0.108	0.365	28	6
Potassium voltage-gated channel subfamily A member 1	KCNA1	0.058	0.313	22	7
Glutamate ionotropic receptor NMDA type subunit 2A	GRIN2A	0.072	0.347	22	6
ATPase plasma membrane Ca2+ transporting 2	ATP2B2	0.101	0.306	18	7
Potassium voltage-gated channel subfamily A member 2	KCNA2	0.041	0.304	18	7
Apolipoprotein E	APOE	0.088	0.329	16	6
Peroxisome proliferator-activated receptor gamma coactivator 1-alpha	PPARGC1A	0.148	0.343	16	6
Sodium voltage-gated channel alpha subunit 1	SCN1A	0.028	0.321	16	6
/	mean	0.033	0.261	7.617	/

### KEGG pathway and GO enrichment analysis

18 KEGG pathways and 25 GO entries were enriched (Tables 2 and 3). Bubble plots of the enrichment analysis results are shown in Fig. 3C, D, respectively. It is speculated that the pathogenesis of SCZ may involve cAMP signaling pathway, neurodegenerative path-multiple diseases, long-term potential difference phenomenon of dopaminergic synapses and other pathways, mainly related to the function of voltage-gated potassium channel active plasma channel. The GO term bar with color gradient of these key targets is displayed in Supplementary materials (Fig. S1).

**Table 2:** KEGG enrichment results of 29 hub targets.

Name	*p*value	Genes	Fold Enrichment	Bonferroni	Benjamini	FDR
hsa04024:cAMP signaling pathway	1.28E−05	CAMK2B, MAP2K1, GRIN2A, MAPK8, BDNF, SST, ATP2B2	11.971	0.002	0.002	0.002
hsa05212:Pancreatic cancer	3.20E−05	RB1, TGFB2, MAP2K1, MAPK8, VEGFA	25.314	0.006	0.003	0.002
hsa05022:Pathways of neurodegeneration - multiple diseases	9.91E−05	CAMK2B, MAP2K1, GRIN2A, MAPK8, BDNF, DCTN1, PLCB1, NEFH	6.467	0.018	0.006	0.004
hsa04728:Dopaminergic synapse	2.75E−04	CAMK2B, GRIN2A, MAPK8, PLCB1, SCN1A	14.575	0.049	0.012	0.009
hsa04720:Long-term potentiation	5.69E−04	CAMK2B, MAP2K1, GRIN2A, PLCB1	22.972	0.098	0.021	0.015
hsa05200:Pathways in cancer	0.001427	CAMK2B, RB1, TGFB2, MAP2K1, MAPK8, PLCB1, VEGFA	5.072	0.228	0.038	0.029
hsa04912:GnRH signaling pathway	0.001479	CAMK2B, MAP2K1, MAPK8, PLCB1	16.549	0.235	0.038	0.029
hsa04933:AGE-RAGE signaling pathway in diabetic complications	0.001823	TGFB2, MAPK8, PLCB1, VEGFA	15.391	0.281	0.041	0.031
hsa04014:Ras signaling pathway	0.002425	MAP2K1, GRIN2A, MAPK8, BDNF, VEGFA	8.152	0.356	0.043	0.033
hsa04725:Cholinergic synapse	0.002585	CAMK2B, MAP2K1, KCNQ5, PLCB1	13.62	0.374	0.043	0.033
hsa04722:Neurotrophin signaling pathway	0.002995	CAMK2B, MAP2K1, MAPK8, BDNF	12.934	0.419	0.043	0.033
hsa04935:Growth hormone synthesis, secretion and action	0.003067	MAP2K1, MAPK8, SST, PLCB1	12.826	0.426	0.043	0.033
hsa04020:Calcium signaling pathway	0.003122	CAMK2B, GRIN2A, ATP2B2, PLCB1, VEGFA	7.604	0.432	0.043	0.033
hsa04926:Relaxin signaling pathway	0.003762	MAP2K1, MAPK8, PLCB1, VEGFA	11.931	0.495	0.049	0.037
hsa05219:Bladder cancer	0.004535	RB1, MAP2K1, VEGFA	28.154	0.561	0.055	0.041
hsa04010:MAPK signaling pathway	0.005877	TGFB2, MAP2K1, MAPK8, BDNF, VEGFA	6.37	0.656	0.063	0.047
hsa05016:Huntington disease	0.006157	MAPK8, BDNF, DCTN1, PLCB1, PPARGC1A	6.287	0.673	0.063	0.047
hsa04934:Cushing syndrome	0.006286	CAMK2B, RB1, MAP2K1, PLCB1	9.93	0.681	0.063	0.047

**Table 3:** GO enrichment results of 29 hub targets.

Category	Term	*p*value	Genes	Fold Enrichment	Bonferroni	Benjamini	FDR
GOTERM_BP_DIRECT	GO0071805~potassium ion transmembrane transport	1.21E−06	KCNV1, KCNC2, KCNA1, KCNA2, SLC17A6, KCNQ5	30.201	0.001	0	0
GOTERM_BP_DIRECT	GO0034765~regulation of ion transmembrane transport	1.26E−06	KCNV1, KCNC2, KCNA1, KCNA2, KCNQ5, SCN1A	29.975	0.001	0	0
GOTERM_BP_DIRECT	GO0099505~regulation of presynaptic membrane potential	1.06E−05	KCNC2, KCNA1, KCNA2, SCN1A	89.26	0.008	0.003	0.003
GOTERM_BP_DIRECT	GO0010629~negative regulation of gene expression	7.20E−05	RB1, TGFB2, GJA1, FBXW7, APOE, VEGFA	12.999	0.055	0.014	0.014
GOTERM_CC_DIRECT	GO:0030424~axon	1.83E−08	TGFB2, MAPK8, KCNC2, BDNF, DCTN1, KCNA2, NEFH, PVALB, SCN1A	17.779	0	0	0
GOTERM_CC_DIRECT	GO:0008076~voltage-gated potassium channel complex	4.21E−06	KCNV1, KCNC2, KCNA1, KCNA2, KCNQ5	43.9	0.001	0	0
GOTERM_CC_DIRECT	GO:0098982 ~ GABA-ergic synapse	5.10E−06	NLGN1, KCNC2, SST, ATP2B2, PLCB1	41.834	0.001	0	0
GOTERM_CC_DIRECT	GO:0045202~synapse	5.74E−06	CAMK2B, NLGN1, MAPK8, KCNC2, SST, KCNA1, KCNA2, PVALB	10.595	0.001	0	0
GOTERM_CC_DIRECT	GO:0043025~neuronal cell body	8.25E−06	TGFB2, DCTN1, SST, KCNA1, APOE, PVALB, SCN1A	13.565	0.001	0	0
GOTERM_CC_DIRECT	GO:0098978~glutamatergic synapse	1.57E−05	GRIN2A, NLGN1, KCNA1, KCNA2, ATP2B2, APOE, PLCB1	12.112	0.003	0	0
GOTERM_CC_DIRECT	GO:0045211~postsynaptic membrane	1.44E−04	GRIN2A, NLGN1, KCNC2, KCNA1, KCNA2	17.869	0.024	0.003	0.003
GOTERM_CC_DIRECT	GO:0030425~dendrite	3.08E−04	NLGN1, KCNC2, BDNF, KCNA1, KCNA2, APOE	9.525	0.05	0.006	0.006
GOTERM_CC_DIRECT	GO:0043194~axon initial segment	3.67E−04	KCNA1, KCNA2, SCN1A	101.596	0.06	0.007	0.006
GOTERM_CC_DIRECT	GO:0005887~integral component of plasma membrane	4.82E−04	KCNV1, GRIN2A, NLGN1, GJA1, KCNC2, KCNA1, KCNA2, ATP2B2, KCNQ5	4.451	0.078	0.008	0.008
GOTERM_CC_DIRECT	GO:0042734~presynaptic membrane	6.68E−04	GRIN2A, KCNC2, KCNA1, KCNA2	22.399	0.106	0.01	0.009
GOTERM_CC_DIRECT	GO:0032809~neuronal cell body membrane	7.05E−04	KCNC2, KCNA2, ATP2B2	73.57	0.112	0.01	0.009
GOTERM_CC_DIRECT	GO:0043005~neuron projection	0.001455	CAMK2B, GRIN2A, DCTN1, KCNA2, SLC17A6	9.663	0.217	0.019	0.018
GOTERM_MF_DIRECT	GO:0099508~voltage-gated ion channel activity involved in regulation of presynaptic membrane potential	4.73E−07	KCNC2, KCNA1, KCNA2, SCN1A	237.555	0	0	0
GOTERM_MF_DIRECT	GO:0005249~voltage-gated potassium channel activity	2.28E−06	KCNV1, KCNC2, KCNA1, KCNA2, KCNQ5	51.037	0	0	0
GOTERM_MF_DIRECT	GO:0005251~delayed rectifier potassium channel activity	1.68E−05	KCNC2, KCNA1, KCNA2, KCNQ5	76.856	0.003	0.001	0.001
GOTERM_MF_DIRECT	GO:0005516~calmodulin binding	2.31E−04	CAMK2B, RGS4, ATP2B2, KCNQ5, PLCB1	15.78	0.034	0.008	0.008
GOTERM_MF_DIRECT	GO:0001540~beta-amyloid binding	2.63E−04	TGFB2, GRIN2A, NLGN1, APOE	30.742	0.039	0.008	0.008
GOTERM_MF_DIRECT	GO:0005267~potassium channel activity	0.001359	KCNA1, KCNA2, KCNQ5	52.968	0.187	0.034	0.033
GOTERM_MF_DIRECT	GO:0019899~enzyme binding	0.002229	DAOA, RB1, MAPK8, APOE, PLCB1	8.596	0.288	0.048	0.046
GOTERM_MF_DIRECT	GO:0042802~identical protein binding	0.002543	CAMK2B, RB1, NLGN1, SST, FBXW7, APOE, PLCB1, PVALB, VEGFA	3.45	0.321	0.048	0.046

### Assessment of molecular markers of SCZ

To further confirm the accuracy of the screened core targets, we subsequently used two new chip data (GSE215985 and GSE87610) to verify the top10 core targets. Firstly, the volcano map and heat map of the differentially expressed genes of the two-chip data were drawn, and the results are displayed in Fig. 1C–F. For the GSE215985 chip, 222 up-regulated genes and 256 down-regulated genes were obtained, respectively (Fig. 1C, D). As well as five up-regulated genes and three down-regulated genes of GSE87610 (Fig. 1E,F).

### Validation of the top 10 core genes

**Fig. 4:**
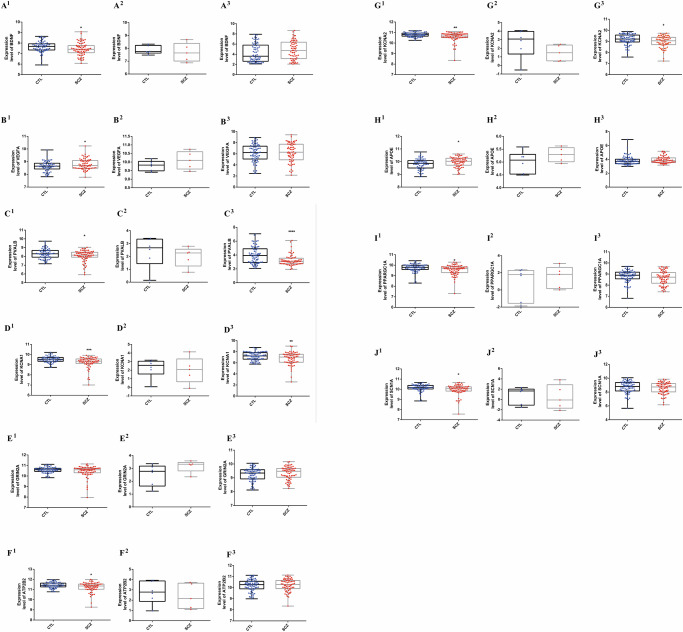
Boxplot of the expression of 10 core targets between the SCZ and CTL groups. Expression results of top 10 differential hub genes between SCZ and CTL were displayed. **A**, **B**, **C**, **D**, **E**, **F**, **G**, **H**, **I** and **J** presents BDNF, VEGFA, PVALB, KCNA1, GRIN2A, ATP2B2, KCNA2, APOE, PPARGC1A and SCN1A, respectively. GSE208338, GSE87610 and GSE215985 is denoted by superscript of **1**, **2**, and **3**, respectively.

The accuracy and reliability of the previously screened key genes was further confirmed by mining the two new GEO data. These boxplots were used to visually illustrate the difference in the trend of expression changes of these 10 targets between healthy and SCZ patients, as well as the trend of expression changes of these targets among in three chip data (GSE208338, GSE215985, and GSE87610). Compared with CON group (healthy control people), indicated above are boxplot pictures that the expression differences of 10 genes in GSE208338 were BDNF (↓, *P* < 0.05), VEGFA (↑), PVALB (↓), KCNA1 (↓), GRIN2A (↑), ATP2B2 (↓), KCNA2 (↓), APOE (↑), PPARGC1A (↓), SCN1A (↓). Furthermore, the expression trends of these genes in SCZ patients in GSE215985 and GSE87610 were consistent with their expression trends in GSE208338. It suggested that the screening results of this study are accurate. The expression levels of the 10 core targets in the subjects of SCZ group and CON group are shown in Fig. 4.

### Molecular docking analysis

After confirming the accuracy of these core targets, the next step was to use molecular docking technology to predict the potential efficacy and possible mechanism of three methyl salicylate glycosides in the treatment of SCZ. The binding affinity between the ligand (3 compounds, MSTG-A, MSTG-B and Gaultherin) and the receptor (10 hub genes) ranged from −4.7 to −109.2 (kcal/mol) (Table 4).

**Table 4:** Docking results of 3 small molecule ligands and 10 receptor proteins.

No.	Key targets	Docking protein name (PDB database)	MSTG-A		MSTG-B		Gaultherin	
			Docking or not	Binding energy	Docking or not	Binding energy	Docking or not	Binding energy
				(kcal/mol)		(kcal/mol)		(kcal/mol)
1	BDNF	1BND	OK	18915125	None	None	OK	9910802
2	VEGFA	6ZFL	None	None	None	None	None	None
3	PVALB	1RK9	OK	150.845	None	None	OK	86.247
4	KCNA1	1EXB	OK	−7.159	OK	−7.117	OK	−6.18
5	GRIN2A	5H8Q	OK	−5.056	OK	−5.455	OK	−4.798
6	ATP2B2	2KNE	OK	−109.172	OK	−109.165	OK	−104.912
7	KCNA2	2R9R	OK	−0.004	OK	−0.038	OK	0
8	APOE	7FCR	OK	−0.003	OK	−0.041	OK	0
9	PPARGC1A	6W9L	OK	−5.884	OK	−6.024	OK	−6.159
10	SCN1A	7DTD	None	None	OK	−3.973	OK	−4.705

In general, we think that the binding energy is less than −4 kcal/mol and has a good affinity. Less than −7 kcal/mol showed a strong affinity. The results of this study show that MSTG-A, MSTG-B and Gaultherin emerged good binding affinity with KCNA1, GRIN2A, ATP2B2 and PPARGC1A, and the interaction of them with ATP2B2 was more stable. They may be the most prominent targets of MSTG-A, MSTG-B and Gaultherin in exerting the therapeutic affection on SCZ. MSTG-B and Gaultherin also presented better binding activity with SCN1A, and the order of docking effect is: Gaultherin > MSTG-B. The docking results indicated that there is a good affinity between the three small molecules and several hub genes. This result suggests that hub genes could be exploited as potential biomarkers of SCZ, and MSTG-A, MSTG-B and Gaultherin might play a role in the treatment of SCZ by regulating these targets.

**Fig. 5:**
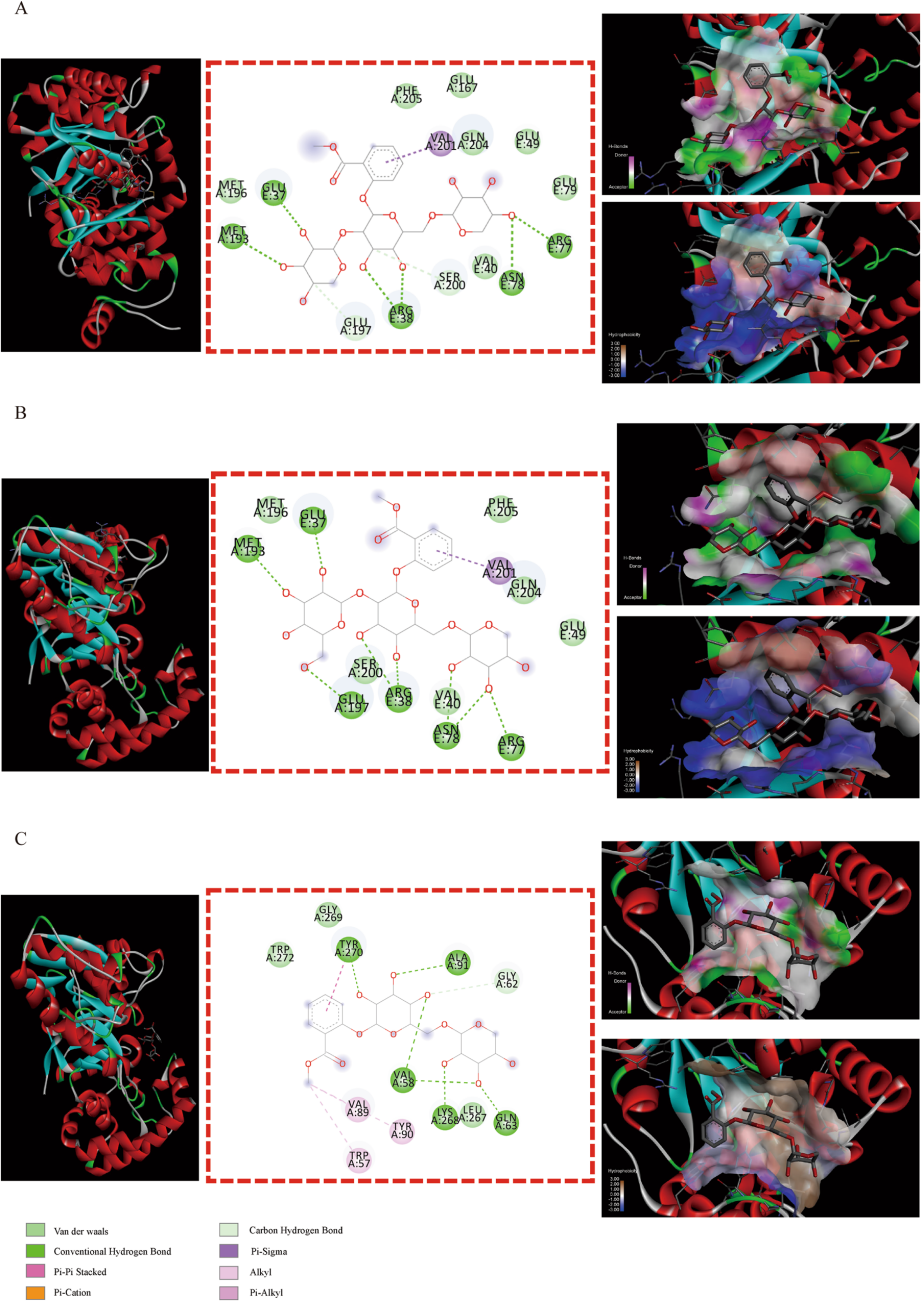
Molecular docking analysis of 3 methyl salicylate glycosides with KCNA1. The overall structure, 3D partial view and 2D binding mode of **A** MSTG-A-KCNA1, **B** MSTG-B-KCNA1, **C** Gaultherin-KCNA1.

**Fig. 6:**
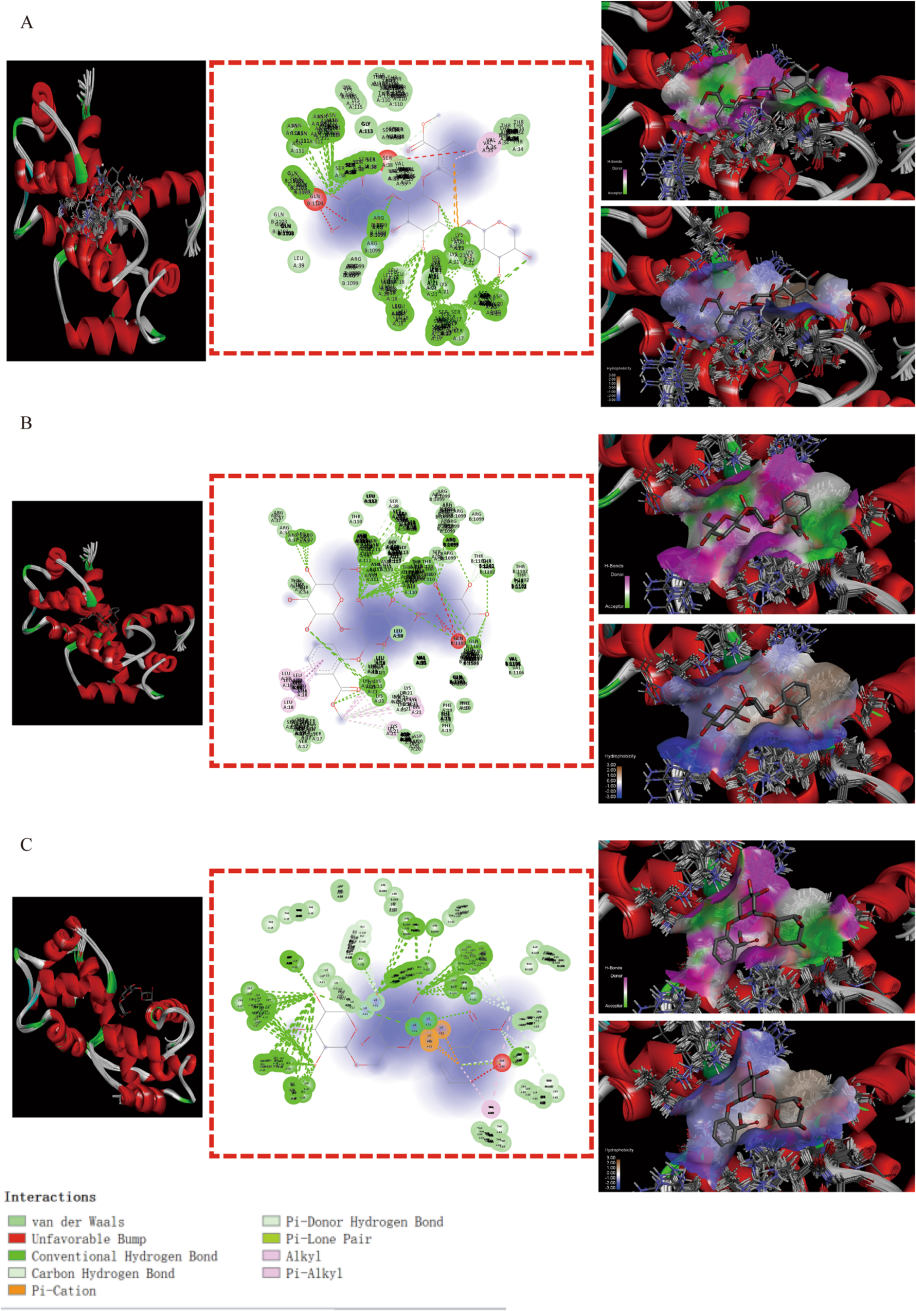
Molecular docking analysis of 3 methyl salicylate glycosides with ATP2B2. The overall structure, 3D partial view and 2D binding mode of **A** MSTG-A-ATP2B2, **B** MSTG-B-ATP2B2, **C** Gaultherin-ATP2B2.

**Fig. 7:**
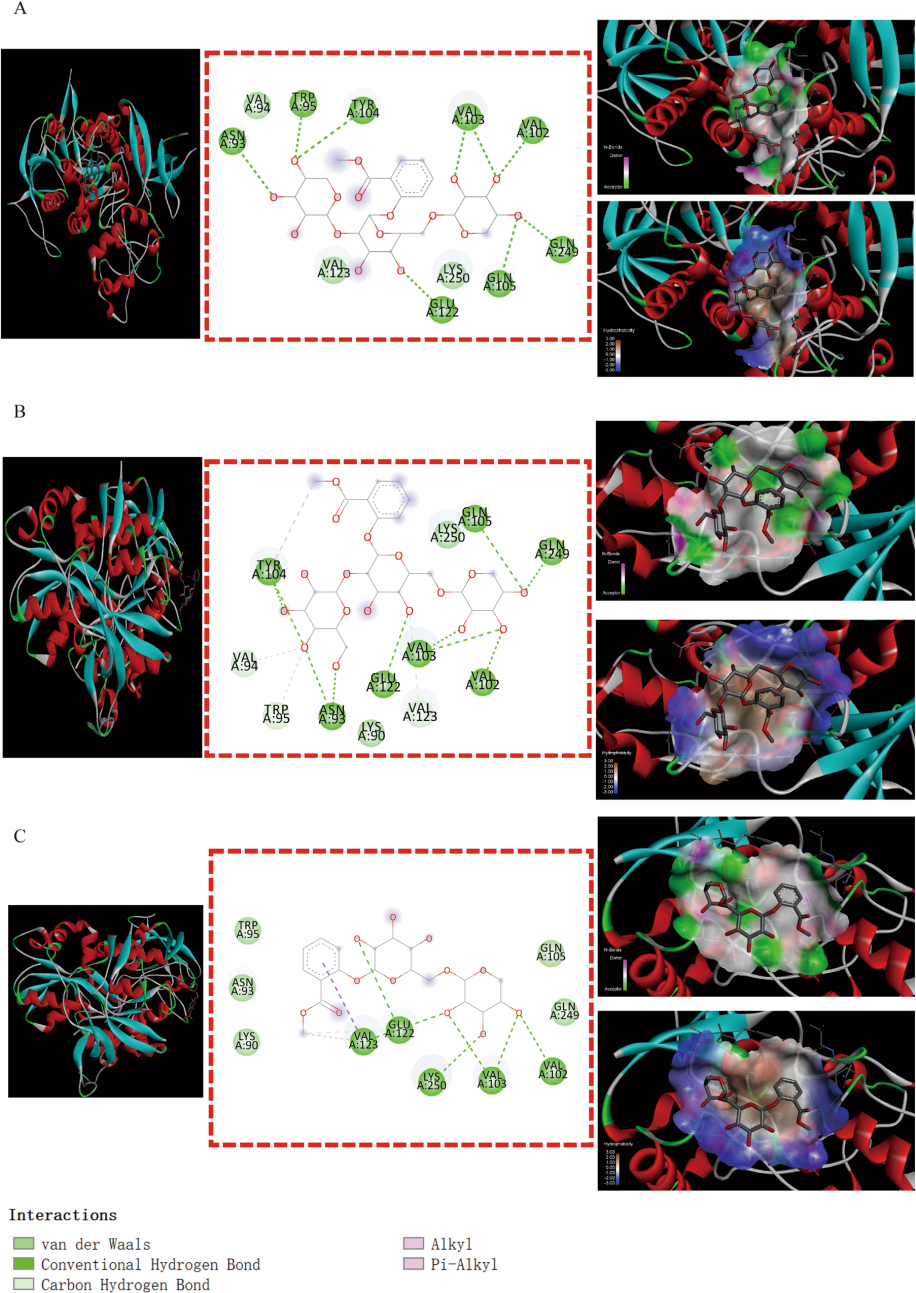
Molecular docking analysis of 3 methyl salicylate glycosides with GRIN2A. The overall structure, 3D partial view and 2D binding mode of **A** MSTG-A-GRIN2A, **B** MSTG-B-GRIN2A, **C** Gaultherin-GRIN2A.

**Fig. 8:**
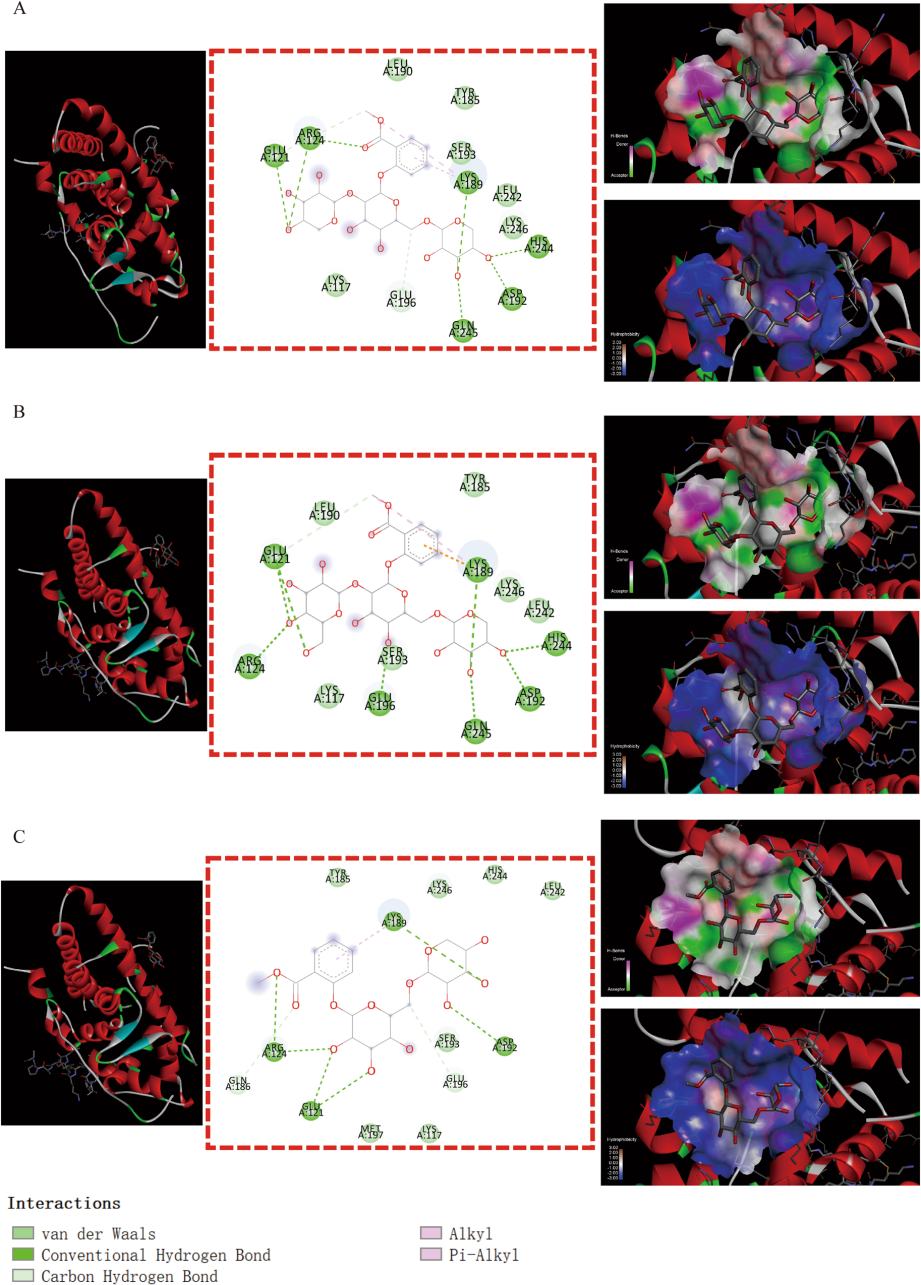
Molecular docking analysis of 3 methyl salicylate glycosides with PPARGC1A. The overall structure, 3D partial view and 2D binding mode of **A** MSTG-A-PPARGC1A, **B** MSTG-B-PPARGC1A, **C** Gaultherin-PPARGC1A.

**Fig. 9:**
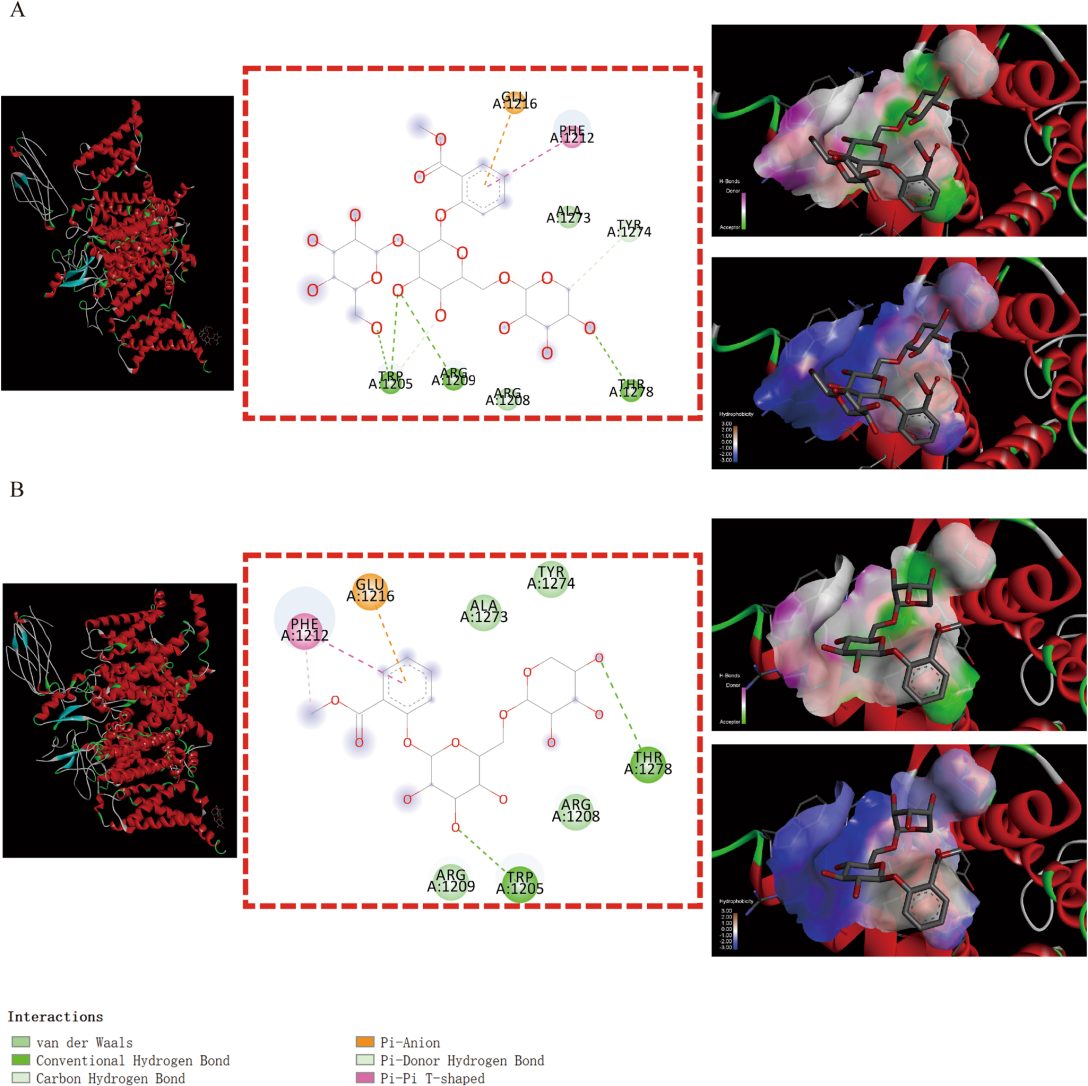
Molecular docking analysis of 2 methyl salicylate glycosides with SCN1A. The overall structure, 3D partial view and 2D binding mode of **A** MSTG-B-SCN1A, **B** Gaultherin-SCN1A.

Here, Figs. 5–9 displayed that the docking results of MSTG-A, MSTG-B, and Gaultherin with several core targets visualized by DS software. As shown in Fig. 5A–C, the results demonstrated that MSTG-A, MSTG-B and Gaultherin exhibited different binding modes with KCNA1 residues through some intermolecular forces (IMFs), which mainly included van der Waals forces, hydrogen bonds, hydrophobic bonds, π-σ, π-π stacking and the other IMFs.

Some binding modes were generated by MSTG-A, MSTG-B and Gaultherin docking with ATP2B2 through the IMFs containing van der Waals forces, hydrogen bonds, hydrophobic bonds, π-σ, π-alkyl groups, alkyl groups, π-single pair electron π-cation, π-donor hydrogen bonds, unfavorable collisions and other IMFs (Fig. 6A–C). For the GRIN2A residues (Fig. 7A–C), the IMFs of MSTG-A, MSTG-B and Gaultherin combing with it were composed of van der Waals forces, hydrogen bonds, hydrophobic bonds, C-H bonds, π-alkyl groups and alkyl groups. MSTG-A, MSTG-B and Gaultherin determined various docking models with PPARGC1A residues under the influence of van der Waals forces, C-H bonds, hydrogen bonds, hydrophobic bonds, π-alkyl groups and alkyl groups (Fig. 8A–C). Except for van der Waals force, C-H bond, hydrogen bond, hydrophobic bond, MSTG-B and Gaultherin generated several combined modes with SCN1A residues through π-anion, π-donor hydrogen bond, π-π T-shaped interaction (Fig. 9A, B).

### MDS analysis

**Fig. 10:**
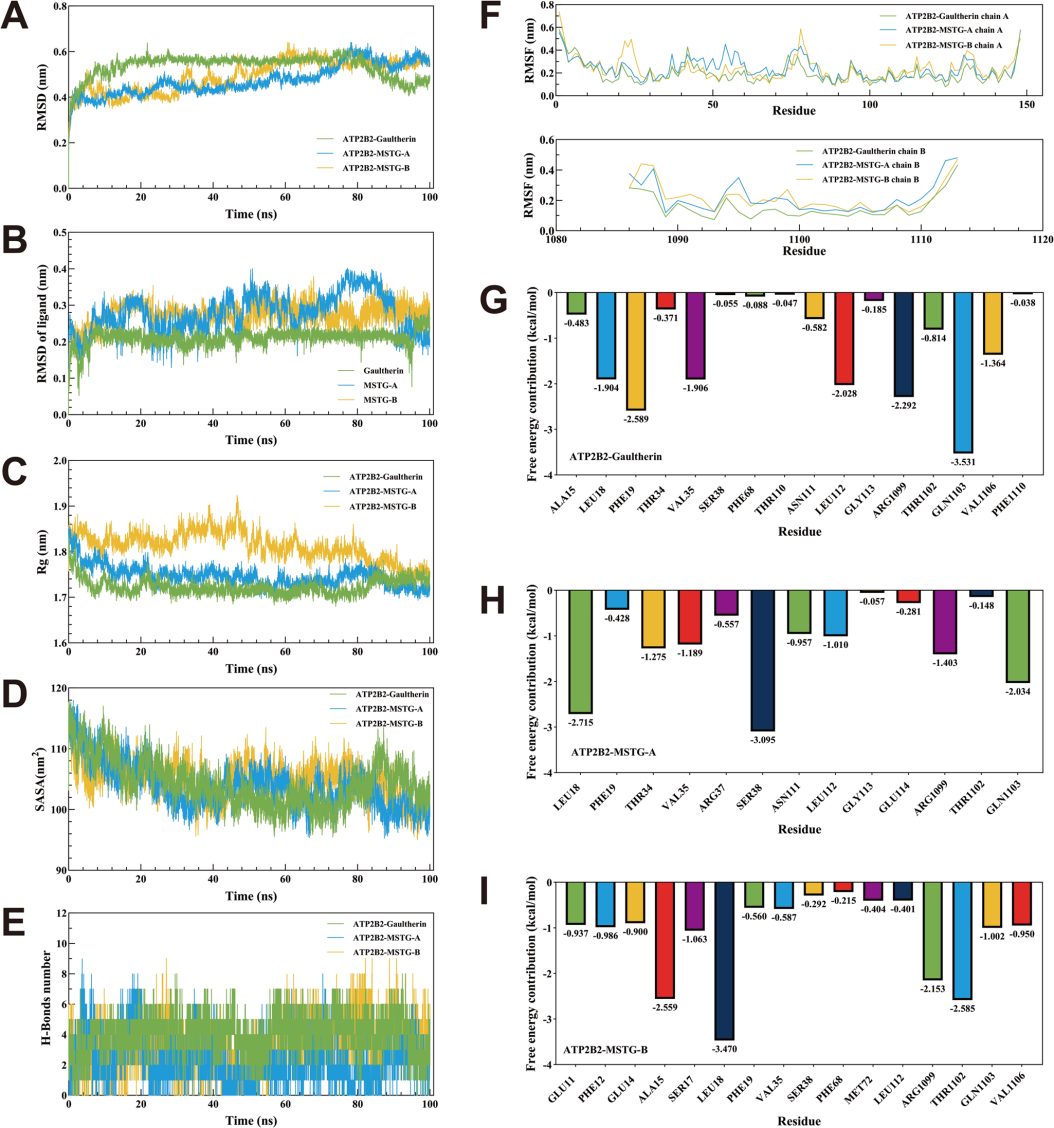
MDS analysis of ATP2B2-Gaultherin/MSTG-A/MSTG-B complex at 100 ns. **A** RMSD curve of ATP2B2-Gaultherin/MSTG-A/MSTG-B complex. **B** RMSD curve of Gaultherin, MSTG-A and MSTG-B. **C** Rg curve of ATP2B2-Gaultherin/MSTG-A/MSTG-B complex. **D** SASA curve of ATP2B2-Gaultherin/MSTG-A/MSTG-B. **E** The number of hydrogen bonds of ATP2B2-Gaultherin/MSTG-A/MSTG-B complex. **F** RMSF plot of ATP2B2-Gaultherin/MSTG-A/MSTG-B complex. **G**–**I** The amino acid breakdown of ATP2B2-Gaultherin/MSTG-A/MSTG-B complex.

MDS was performed to verify the binding abilities between three small molecule components and one of the key target proteins with optimal binding abilities in molecular docking. MSTG-A, MSTG-B, Gaultherin and ATP2B2 protein (PDB ID: 2KNE) were chosen for MDS because they showed the strongest binding force in molecular docking procedures. Their results of molecular docking were displayed in Fig. S2 (Supplementary materials). The value of RMSD was utilized for assessing the balance of the MDS system. In Fig. 10A, the MSTG-A/ATP2B2, MSTG-B/ATP2B2, Gaultherin/ATP2B2 protein complex have been stabilized with little fluctuation in the range of 20–80 ns, the average RMSD value of these complexes was approximately 0.55 nm. It revealing that the binding between 3 compounds and ATP2B2 are extremely stable, particularly Gaultherin (Fig. 10B). The Rg curve of the ATP2B2 and Gaultherin/MSTG-A complex remained essentially stable throughout, and was superior to that of MSTG-B (As shown in Fig. 10C). The SASA curve of the ATP2B2-Gaultherin/MSTG-A/MSTG-B complex showed an overall decreasing trend (Fig. 10D). Fig. 10E showed that the number of hydrogen bonds of Gaultherin/MSTG-A/MSTG-B-ATP2B2 complex varied in the range of 2–8, 2–9 and 2–9, respectively, during the simulation of 100 ns. As exhibited in Fig. 10F, RMSF analysis showed that there was no significant difference in the flexibility of amino acid residues of ATP2B2 after binding with Gaultherin, MSTG-A and MSTG-B, indicating that Gaultherin, MSTG-A and MSTG-B had little effect on the amino acid flexibility of ATP2B2. A more negative free energy contribution value for a residue indicates a greater contribution of that residue to ligand binding. The highest contribution residue of Gaultherin/MSTG-A/MSTG-B-ATP2B2 was GLN1103, SER38 and LEU18, respectively, as shown in Fig. 10G–I.

**Fig. 11:**
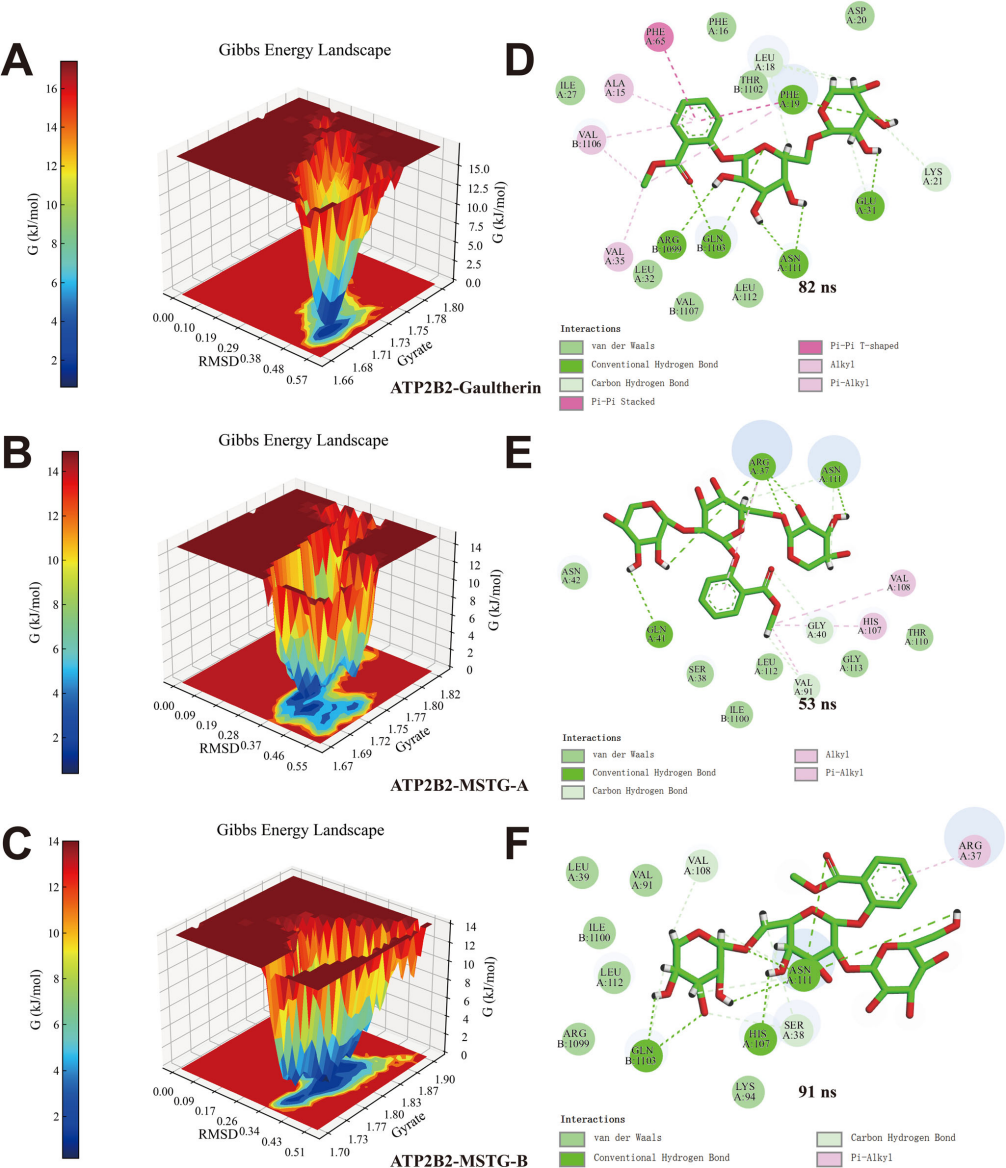
GFE analysis of ATP2B2-Gaultherin/MSTG-A/MSTG-B complex. **A**–**C** The GFE landscape of ATP2B2-Gaultherin/MSTG-A/MSTG-B complex. **D**–**F** The 2D interaction diagram of ATP2B2-Gaultherin/MSTG-A/MSTG-B complex at the lowest GFE point.

The built-in scripts “g_sham” and “xpm2txt.py” of Gromacs v2022.03 software were used to calculate GFE according to RMSD and Rg values of ATP2B2-Gaultherin/MSTG-A/MSTG-B complex. The GFE 3D topography was obtained by the values of RMSD, Rg and GFE. As shown in Fig. 11A–C, the GFE 3D morphology of ATP2B2-Gaultherin/MSTG-A/MSTG-B complex all has a single and sharp lowest energy region. The 7, 5 and 7 hydrogen bonds formed in the ATP2B2-Gaultherin complex (Fig. 11D), the ATP2B2-MSTG-A complex (Fig. 11E), and the ATP2B2-MSTG-B (Fig. 11F), respectively. It was reckoned that these hydrogen bonds help maintain the stability of the three complexes. 2D interaction diagrams of ATP2B2-Gaultherin/MSTG-A/MSTG-B complex at the lowest GFE energy moment were displayed in Fig. 11D–F. Through MM-PBSA method, BFE was calculated using the last 20 ns of stable RMSD trajectorie (Table 5). The total BFE values of ATP2B2-Gaultherin/MSTG-A/MSTG-B complex were −39.73, −26.61 and −41.61 kcal/mol, respectively. Their van der Waals force (ΔVDWAALS), electrostatic force (ΔE_elec_) and gas-phase energy (ΔE_gas_) were all favored the stability of the 3 small molecule-protein complexes system. The results of MDS were in agreement with the molecular docking results, further supporting the excellent potential of methyl salicylate glycosides for the treatment of SCZ.

**Table 5:** The BFE analysis of the ATP2B2-Gaultherin/MSTG-A/MSTG-B complex (kcal/mol).

Energy contributions	ATP2B2-Gaultherin	ATP2B2-MSTG-A	ATP2B2-MSTG-B
Δ*VDWAALS*	−49.07	−43.74	−55.92
Δ*E*_*elec*_	−36.19	−12.6	−38
Δ*E*_*surf*_	−7.06	−6.05	−7.42
Δ*G*_*gas*_	−85.26	−56.34	−93.93
Δ*G*_*solvation*_	45.52	29.73	52.32
Δ*G*_*Bind*_	−39.74	−26.61	−41.61

Δ*VDWAALS*, Δ*E*_*elec*_, Δ*E*_*surf*_, Δ*G*_*gas*_ and Δ*G*_*solvation*_ represent the energy component of van der Waals force, electrostatic force, surface, gas phase and solvation, respectively. The gas phase energy (Δ*G*_*gas*_) is usually derived from the Molecular mechanical (MM) energy in the force field, which includes the contribution of van der Waals and Coulomb forces. Negative Δ*G*_*gas*_ values indicated favorable dissolution in all polymer environments, with MSTG-B exhibiting the most favorable dissolution. The free energy of solvation (Δ*G*_*solvation*_) is calculated using the implicit solvent model. Δ*G*_*Bind*_ is the sum of the gas phase energy (Δ*G*_*gas*_) and the free energy of solvation (Δ*G*_*solvation*_), which is an important indicator of the affinity of the interaction.

## Discussion

There is ongoing skepticism regarding the etiology and management of SCZ in China and globally. Given its complexity and unique nature, SCZ is exacerbating the economic strain on society, warranting increased scrutiny in this area. The adverse effects of current pharmacological treatments cannot be disregarded, underscoring the pressing need for research into novel drug therapies for SCZ. It is imperative to delve deeply into exploring and uncovering new strategies and approaches for addressing this challenge. TCM/ECM a valuable asset of Chinese culture with a rich history of application, has shown significant efficacy in the treatment of complex and challenging diseases. Utilizing big data mining techniques to identify effective ingredients for the treatment of SCZ within TCM/ECM represents a promising approach. This study adopted public databases, visualization software, bioinformatics mining technology, network pharmacology, and molecular docking technology to jointly investigate the pathogenesis of SCZ and the potential therapeutic effects and mechanisms of action of MSGs components for SCZ.

Due to the high heritability of SCZ, deciphering the genetic susceptibility factors would lead us to a better understanding of the genetic basis of schizophrenia. We have identified potential biomarkers for SCZ by GEO database. 10 hub genes were identified, including BDNF, VEGFA, PVALB, KCNA1, GRIN2A, ATP2B2, KCNA2, APOE, PPARGC1A and SCN1A. They play an important role in the pathogenesis and treatment mechanism of SCZ. Brain-derived neurotrophic factor, abbreviated as BDNF, is a protein with important regulatory effects on neuronal growth, differentiation and function, which is also involved in the regulation of neuronal synaptic plasticity, and the abnormality of synaptic plasticity is one of the important pathological features of SCZ^35,36^. Studies have shown that variations in the BDNF are associated with the risk, severity of cognitive performance and negative symptoms of SCZ^37,38^. The expression level of BDNF was found to be decreased in patients with SCZ, and was associated with disease severity and cognitive impairment^37,39^. In recent years, many studies have found that the VEGFA gene may be related to the pathogenesis of SCZ, providing new insights into the understanding of the etiology of SCZ and may provide clues to future treatment strategies^40^. It has been proposed that the detection of VEGF in blood may be a feasible way to distinguish MDD and SCZ^41^. Deficits of brain parvalbumin (PV) are a consistent finding in schizophrenia and models of psychosis^42^. Chemical dysregulation of synaptic transmission in two types of GABAergic neurons (PVALB and LAMP5) has been observed^43^. PVALB, as an abnormal PV gene in the brain, was used as key genes to construct the risk prediction model for early diagnosis of SCZ through a joint machine learning algorithm^44^. KCNA1 (potassium voltage-gated channel, shaker-related subfamily, alpha1) encodes proteins involved in various biological processes, including neurogenesis and cell adhesion, which are implicated in the development and progression of SCZ^45,46^. *Nature* published that the glutamate receptor subunit GRIN2A is one of the risk genes contributing to glutamatergic involvement in SCZ^47,48^. As a fine-mapped candidate, GRIN2A was enriched for genes associated with rare disruptive coding variants in people with SCZ, whose biological processes are relevant to the pathophysiology of SCZ^49^.

ATP2B2 was identified as a risk gene for SCZ, expressed in multiple brain tissue types, which was involved in intracellular calcium homeostasis and predicted to be intolerant to loss-of-function and missense variants^50,51^. ATP2B2 shows highly suggestive evidence for deleterious missense variants in SCZ cases (*p* = 0.000072)^50^, and has been highlighted as potentially relevant to risperidone activity^52^.

KCNA2 (potassium voltage-gated channel subfamily A member 2) are formed in most brain structures, reflecting the marked variation in severity observed in many ion channel disorders^53,54^. Currently, de novo mutations of KCNA2 gene, have been confirmed to cause a new molecular entity within the epileptic encephalopathies, which could cause either a dominant-negative loss-of-function or a gain-of-function of the voltage-gated K^+^ channel KCNA2^55^. It was assayed that APOE (apolipoprotein E) gene polymorphisms might be involved in the pathogenesis of SCZ^56^. APOE is associated with metabolic processes in the brain and plays a critical role in the synapse, affecting on lipid homeostasis, myelin maintenance and integrity, making it an attractive candidate in the pathogenesis of SCZ^57,58^. PPARGC1A has been recognized as a leading candidate gene for schizophrenia (SCZ) through genome-wide association studies, and has been found to play a role in the postnatal brain development in individuals with SCZ^59^. As a key regulator of adipogenesis, the PPARGC1A gene is involved in the control of mitochondrial functions^60^, with higher levels observed in younger individuals with SCZ but not in monkeys exposed to antipsychotic medications^61^.

SCN1A (encoding the α subunit of the type I voltage-gated sodium channel), exhibited decreased allele-dependent activation differences in brain regions typically involved in working memory processes, suggesting that it has plays a key role in human short-term memory^62^. The association of SCN1A mutation, childhood SCZ and autism spectrum disorder without epilepsy was first reported in *Psychiatry Research*^63^. The selective activators of the sodium channel were presented that they may hold therapeutic potential for diseases such as SCZ, epilepsy and Alzheimer’s disease^64,65^. These 10 targets are referred to the occurrence and development of SCZ from different angles and different levels, and have important potential development value for the development of new drugs and targeted therapy in the future.

These hub targets were further analyzed and summarized to better understand the target functions associated with SCZ. The present study demonstrated that the pathogenesis of SCZ may be mainly related to the down-regulation of BDNF, PVALB and KCNA1 expression in the body, together with the up-regulation of VEGFA expression. These results of the two GSE data exhibited the same trend of top ten genes, which is regarded as the further validation illustrated the analysis accuracy of GSE208338 in this paper. Considering the limitations of bioinformatics analysis, these 10 key targets obtained were validated using two additional GSE datasets (GSE87610 and GSE215985). The results showed that the expression trends of the 10 genes in these three GSE datasets were consistent, and were reported in the literature on pathogenesis and diagnosis and treatment mechanisms of SCZ, indicating the reliability of the bioinformatics analysis method in this study. Of course, there are also shortcomings, which will be thoroughly studied and examined in our subsequent research work.

KEGG pathway enrichment analysis demonstrated that these genes participated in the regulation of signaling pathways such as cAMP signaling pathway (hsa04024)^66^, Pancreatic cancer(hsa05212)^67^, Pathways of neurodegeneration - multiple diseases (hsa05022)^68,69^, Dopaminergic synapse (hsa04728)^70^, Long-term potentiation (hsa04720)^71–73^. Similar conclusions can be drawn from much of the literatures, suggesting that these pathways have good potential and correlation with the occurrence and development process of SCZ^66,74^. Of particular note are the studies that have shown one in twelve patients with pancreatic cancer has a pre-existing psychiatric disorder^66^, indicating that the relationship of pancreatic cancer pathway or the others cancer pathway and SCZ need to be concerned in the future. Potassium ion transmembrane transport refers to the movement of potassium ions across the cell membrane, which is crucial for various physiological processes, including the generation of nerve impulses and the maintenance of neuronal excitability^75^. Potassium ion transmembrane transport was identified by GO enrichment analysis, which was considered as an important BP of these hub genes. In SCZ, abnormalities in neurotransmitter systems, brain structure, and neuronal signaling have been observed, and dysfunctions in potassium channels have also been implicated. Potassium ion transmembrane transport can potentially influence cAMP signaling through its effects on neuronal excitability and neurotransmission^76,77^. It is worth mentioning that in our previous study, we performed transcriptome analysis monocytes from the peripheral blood of patients with SCZ and healthy controls (HC). The pathways of Nervous system, Signal transduction, Signaling molecules and interaction, Cancer: overview, Energy metabolism, Transport and catabolism, Immune system, Metabolism of cofactors and vitamins, together with cell migration, regulation of transport, calcium ion sensor activity, cAMP response element binding and other functions were enriched by KEGG and GO enrichment analysis of differentially expressed genes between SCZ and HC groups. This part of the work has not been published at present, but it provides important support and corroboration for the results of this study to a certain extent, suggesting the reliability of the results of this study. There is evidence to suggest that disturbances in potassium ion transmembrane transport may contribute to the development and progression of SCZ. It is important to note that the relationship between these two factors is not yet fully understood and further research is needed to establish a definitive link. Further research is needed to unravel the complex interactions between potassium ion transport, genetic factors, and other physiological and neurochemical abnormalities associated with SCZ^78^. The administration of certain drugs that target potassium channels has been found to affect symptoms associated with SCZ. This is also a focus of our next line of research.

MSTG-A, MSTG-B and Gaultherin isolated from Dianbaizhu, which is an ECM with the effect of “*Qufengchushi*, *Qingrejiedu*, *Huoxuehuayu*”^13,17^. They carried out good anti-inflammatory and analgesic activity with a structural parent nucleus similar to aspirin^16,23^. As we know, anti-inflammatory and immunomodulatory therapeutic strategies have been a major focus in the treatment of SCZ in recent years^11,79,80^. As a classic non-steroidal anti-inflammatory drug, aspirin has also attracted much attention in psychiatric treatment, and its efficacy is still in the process of evaluation and practice, indicating that the components have high application prospects and research value^7,8,10^. In this study, MSTG-A, MSTG-B and Gaultherin have obvious potential efficacy and advantages in the treatment of SCZ. The potential mechanism of the three methyl salicylate glycosides in the treatment of SCZ may be associated with the expression of KCNA1, GRIN2A, ATP2B2 and PPARGC1A. MSTG-B and Gaultherin may also be involved in the regulation of SCN1A protein expression. The results of the present study showed encouraging results of chemical components of TCM/ECM treatment for SCZ patients. In addition, MDS results are consistent with the findings of molecular docking that Gaultherin, MSTG-A, MSTG-B can bind well with ATP2B2, which is the most critical gene in them against SCZ. It provides an important reference and scientific basis for the in-depth exploration and clinical promotion of methyl salicylate glycosides for the alleviation of SCZ.

It is imperative to recognize the constraints of this research. While the findings of this investigation show promise and may contribute to the advancement of novel pharmaceuticals for SCZ, the molecular docking methodology employed to assess the potential regulatory impact of three MSGs compounds on SCZ exhibited limitations that necessitate validation at the cellular and/or animal experimental level. Owing to time constraints and the ongoing development of the animal platform, additional experimental validation of the molecular docking outcomes in vivo and in vitro is yet to be conducted in this study. This is also among our forthcoming priorities, as we are diligently engaged in advancing pertinent verification research. We intend to augment this endeavor in due course. Another constraint pertains to the GEO datasets examined in the present study. The GEO serves as a publicly accessible repository containing a vast collection of gene expression data, thereby representing a significant asset for bioinformatics investigations. Nevertheless, the utilization of GEO data for information generation analysis is subject to several constraints, encompassing disparities in data quality, variations in data standardization techniques, absence of clinical data, data heterogeneity, and inherent limitations of the analytical methodologies employed. Despite our deliberate constraints in the selection of GEO datasets, our study was constrained by the lack of comprehensive inclusion of datasets. The next study will prioritize improved screening of representative data sets through the integration of machine learning and other analytical techniques, followed by thorough analysis. Given the limitations of the analysis method in elucidating the intricacies and biological relevance of the data, we propose enhancing the analysis by amalgamating data from various omics disciplines and integrating clinical data. Multiple data sets and experimental validation methods will be employed to ensure the robustness of each finding in our further investigations.

## Conclusions

In summary, we performed bioinformatic analysis, conduction of PPI network, and elucidation of topological features of the hub genes associated with SCZ were conducted using various databases and visualization software. The identification and validation of genes potentially in the pathogenesis of SCZ were successfully accomplished. Additionally, the prediction of binding affinity between three MSGs and the top 10 targets of SCZ was preliminarily accomplished through molecular docking techniques. The potentially important regulatory values of MSTG-A, MSTG-B and Gaultherin on ATP2B2 were successfully verified by MDS, which corroborating the molecular docking results. The potential value of ATP2B2 was This research offers new insights into advancing the developmental trajectory of SCZ and elucidating its pathogenesis, as well as excavating and exploring of new potential therapeutic targets for SCZ. Subsequent steps involve conducting expeditious in vivo and in vitro validate the findings of this study and facilitate the utilization and translation of MSTG-A, MSTG-B and Gaultherin.

### Supplementary information

Figure S1

Figure S2

Table S1 Detailed information of PPI network of 160 common targets

## Data Availability

The original contributions presented in this study are included in the article/Supplementary Material, further inquiries can be directed to the corresponding authors.
