# Supplementary figures and images for: Exploration on the potential efficacy and mechanism of methyl salicylate glycosides in the treatment of schizophrenia based on bioinformatics, molecular docking and dynamics simulation
Source: Schizophrenia (Heidelb). 2024 Jul 17;10(1):64. doi: 10.1038/s41537-024-00484-y (PMC11255270; doi:10.1038/s41537-024-00484-y)

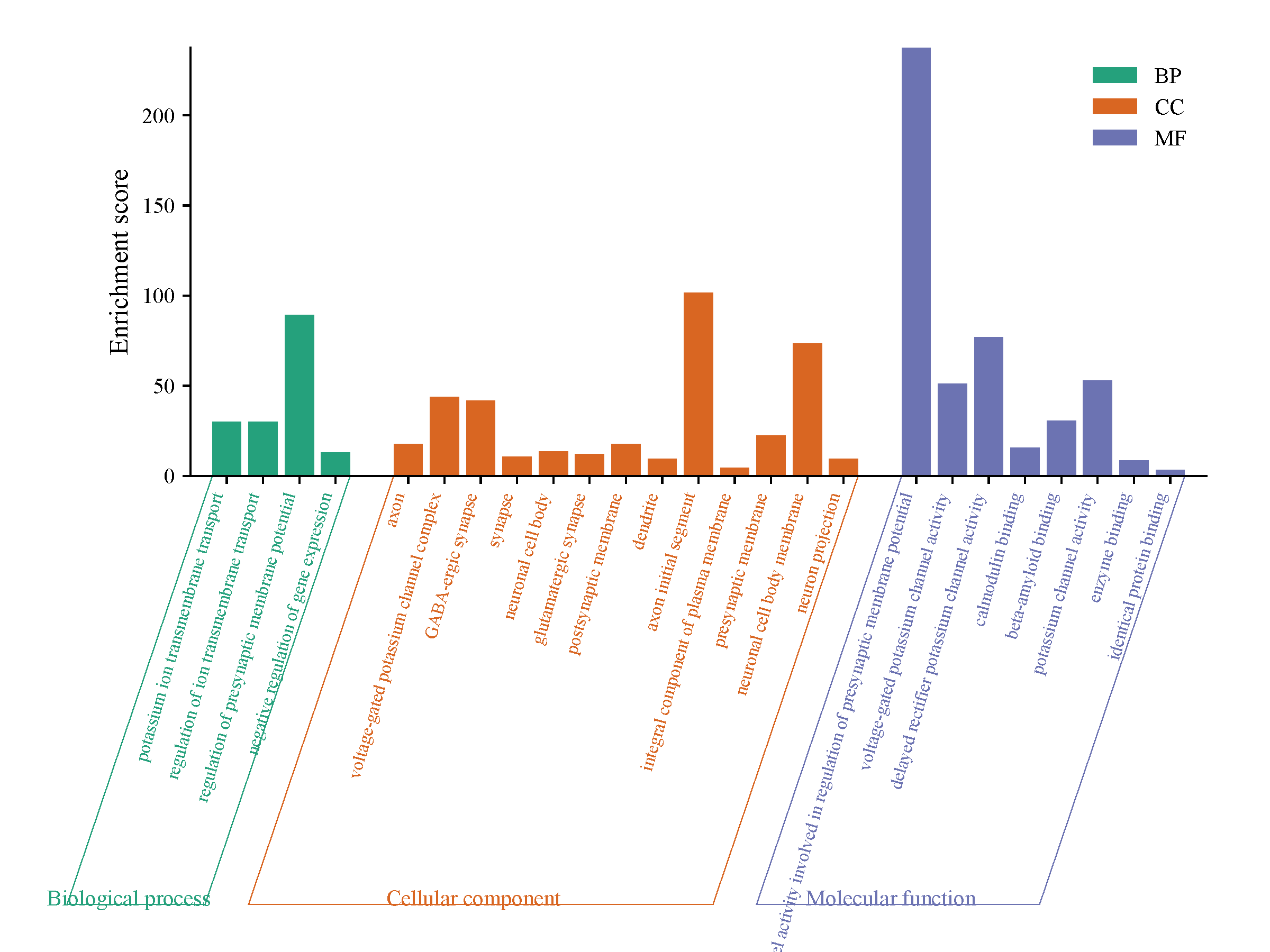

Supplement: Supplementary file 1 — Figure S1 [file 41537_2024_484_MOESM1_ESM.png]

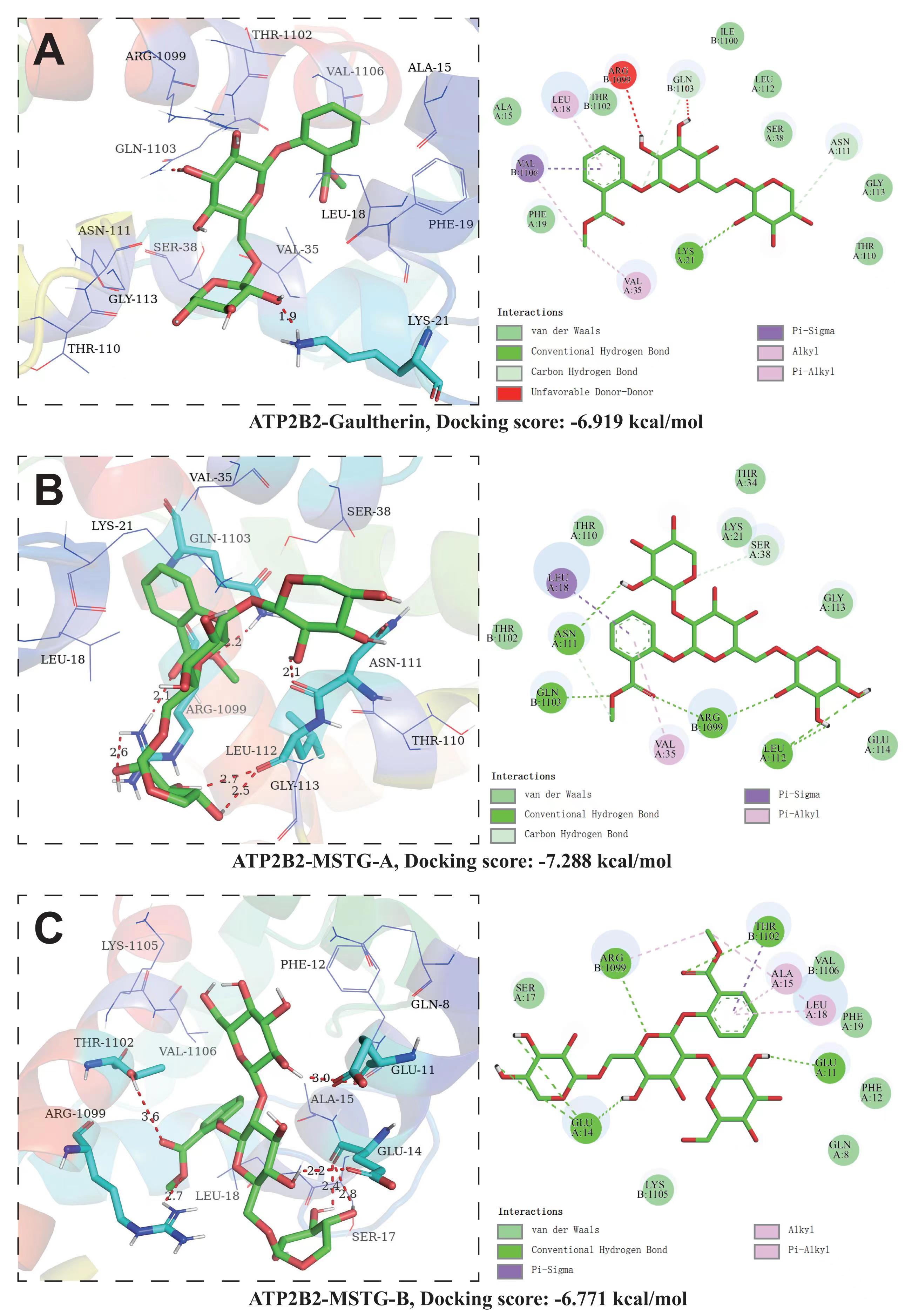

Supplement: Supplementary file 2 — Figure S2 [file 41537_2024_484_MOESM2_ESM.jpg]
